# Right-hand-side updating for fast computing of genomic breeding values

**DOI:** 10.1186/1297-9686-46-24

**Published:** 2014-04-03

**Authors:** Mario PL Calus

**Affiliations:** 1 Animal Breeding and Genomics Centre, Wageningen UR Livestock Research, 6700 AC Wageningen The Netherlands

## Abstract

**Background:**

Since both the number of SNPs (single nucleotide polymorphisms) used in genomic prediction and the number of individuals used in training datasets are rapidly increasing, there is an increasing need to improve the efficiency of genomic prediction models in terms of computing time and memory (RAM) required.

**Methods:**

In this paper, two alternative algorithms for genomic prediction are presented that replace the originally suggested residual updating algorithm, without affecting the estimates. The first alternative algorithm continues to use residual updating, but takes advantage of the characteristic that the predictor variables in the model (i.e. the SNP genotypes) take only three different values, and is therefore termed “improved residual updating”. The second alternative algorithm, here termed “right-hand-side updating” (RHS-updating), extends the idea of improved residual updating across multiple SNPs. The alternative algorithms can be implemented for a range of different genomic predictions models, including random regression BLUP (best linear unbiased prediction) and most Bayesian genomic prediction models. To test the required computing time and RAM, both alternative algorithms were implemented in a Bayesian stochastic search variable selection model.

**Results:**

Compared to the original algorithm, the improved residual updating algorithm reduced CPU time by 35.3 to 43.3%, without changing memory requirements. The RHS-updating algorithm reduced CPU time by 74.5 to 93.0% and memory requirements by 13.1 to 66.4% compared to the original algorithm.

**Conclusions:**

The presented RHS-updating algorithm provides an interesting alternative to reduce both computing time and memory requirements for a range of genomic prediction models.

## Background

Many models have been suggested for genomic prediction (for a review: see [1]). The computing time required to estimate SNP (single nucleotide polymorphism) effects varies considerably between models, e.g. [2]. Computing time depends both on the number of SNPs used and the number of animals in the training dataset. The latter is rapidly increasing, exceeding 15 000 animals in some cases, e.g. [3]. The number of SNPs used is also increasing rapidly with the availability of high-density SNP panels in cattle with 648 874 and 777 962 SNPs [4] and recently, investigations on the use of whole-genome sequence data in genomic prediction have been reported [5,6]. These developments emphasize an increasing need to improve the efficiency of genomic prediction models in terms of computing time and memory requirements. To overcome computing limitations, some fast implementations have been reported for genomic prediction models such as BayesA [7], BayesB [8,9] and Bayesian Lasso [10]. At the same time, it has been suggested that variable selection methods such as BayesB are required to make optimal use of whole-genome sequence data in genomic prediction [6]. The number of reports that compare the fast implementation of such variable selection methods to the Markov chain Monte Carlo (MCMC) based counterparts has thus far been limited, and all of the aforementioned studies were based on simulated data with a limited number of simulated QTL. To enable the comparison of these fast methods to their MCMC based counterparts in real datasets with whole-genome sequence data, efficient implementations of MCMC genomic prediction models are also required.

Genomic prediction models can be classified into those that involve implicit estimation of SNP effects (using genomic relationships), e.g. [11], and those that involve explicit estimation of SNP effects [12]. Genomic prediction models that explicitly estimate SNP effects, commonly perform regression with SNP genotypes as predictor variables [1], coded as 0,1,2 or -1,0,1, referring respectively to the homozygous, heterozygous, and the alternative homozygous genotype. The characteristic that the predictor variables can take only three possible values provides an interesting opportunity to reduce the computing time of algorithms to estimate SNP effects.

The objective of this paper was to describe two efficient algorithms to estimate SNP effects that take advantage of the characteristic that each predictor variable (SNP genotype) can take only three different values. The efficiency of the two algorithms is compared in terms of memory and computing time requirements to that of a commonly used algorithm that is based on residual updating.

## Methods

### Updating schemes to estimate SNP effects

In general, the efficiency of algorithms to estimate SNP effects can be improved by avoiding redundant computations. The general conditional genomic prediction model to estimate SNP effects for locus *j*, is:

$$y_{j}^{*}=1\mu +x_{j}a_{j}+e,$$

where $y_{j}^{*}$ is a vector with conditional phenotypes for SNP *j*, **1** is a vector of 1’s, *μ* is the overall mean, **x**_*j*_ is a vector with SNP genotypes at locus *j*, *a*_*j*_ is the allele substitution effect for locus *j*, and **e** is a vector of residuals. Note that elements of **x**_*j*_ could be simply equal to 0, 1, or 2, or take any other value. I.e., elements of **x**_*j*_ could be scaled and centred, such that they take the following values: $\frac{0-2p_{j}}{\sqrt{2p_{j}\left(1-p_{j}\right)}}$, $\frac{1-2p_{j}}{\sqrt{2p_{j}\left(1-p_{j}\right)}}$ or $\frac{2-2p_{j}}{\sqrt{2p_{j}\left(1-p_{j}\right)}}$, where *p*_*j*_ is the frequency of the allele at locus *j* for which the homozygous genotype is coded as 2. Such scaling of the genotype coding is reported to have some numerical advantages when using MCMC methods [13]. Conditional phenotypes ($y_{j}^{*,l+1}$) for SNP *j* in iteration *l* + 1 are defined as phenotypes corrected for estimated effects at all other SNP loci, as [14]:

(1) $$y_{j}^{*,l+1}=y-X_{1:j-1}{\hat{a}}_{1:j-1}^{l+1}-X_{j+1:n}{\hat{a}}_{j+1:n}^{l}-\mu ,$$

where *n* is the number of SNPs included in the model and **X** is a matrix that stores all genotypes. The conditional mean of the allele substitution effect (${\hat{a}}_{j}^{l+1}$) for locus *j* in iteration *l* + 1 is obtained as follows:

(2) $$\begin{array}{l} {\hat{a}}_{j}^{l+1}=\frac{x_{j}^{'}\left(y-X_{1:j-1}{\hat{a}}_{1:j-1}^{l+1}-X_{j+1:n}{\hat{a}}_{j+1:n}^{l}-\mu \right)}{x_{j}^{'}x_{j}+\lambda_{j}}\\ \;=\frac{x_{j}^{'}y_{j}^{*,l+1}}{x_{j}^{'}x_{j}+\lambda_{j}}, \end{array}$$

where $\lambda_{j}=\frac{\sigma_{e}^{2}}{\sigma_{a_{j}}^{2}}$, $\sigma_{e}^{2}$ is the residual variance, and $\sigma_{a_{j}}^{2}$ is the variance associated with locus *j*. Note that $\sigma_{a_{j}}^{2}$ in equation (2) can be estimated in several ways, as done in well-known models such as BayesA and BayesB [12], BayesC [15] or Bayesian Stochastic Search Variable Selection [16,17], or can be assumed to be known as in RR-BLUP [12,18]. Note that equation (2) gives the value required in a Gauss-Seidel algorithm to compute BLUP estimates for the allele substitution effects, while it gives the mean of the conditional posterior density if a Bayesian model is used to estimate the allele substitution effects.

Using residual updating, the conditional phenotypes in equation (1) in iteration *l* + 1, $y_{j}^{*,l+1}$, can be more efficiently computed as [14]:

(3) $$y_{j}^{*,l+1}=e_{j}^{l+1}+x_{j}{\hat{a}}_{j}^{l},$$

where $e_{j}^{l+1}\;$ contains the current residuals, i.e.:

$$e_{j}^{l+1}=y-X_{1:j}{\hat{a}}_{1:j}^{l+1}-X_{j+1:n}{\hat{a}}_{j+1:n}^{l}-\mu .$$

Using residual updating, the conditional mean of the allele substitution effect (${\hat{a}}_{j}^{l+1}$) in iteration *l* + 1 can then be obtained per locus as follows [14]:

(4) $${\hat{a}}_{j}^{l+1}=\frac{x_{j}^{'}e_{j}^{l+1}+x_{j}^{'}x_{j}{\hat{a}}_{j}^{l}}{x_{j}^{'}x_{j}+\lambda_{j}}.$$

Considering that, in each iteration, allele substitution effects must be estimated for *n* loci, using phenotypes of *m* individuals, computing all conditional phenotypes using equation (1) requires *mn*(*n*-1) multiplications and *mn*(*n*-1) subtractions, whereas equation (3) requires only *mn* multiplications and *mn* summations. After calculating ${\hat{a}}_{j}^{l+1}$, the residual updating step is finalized by updating all residuals such that they can be used to compute conditional phenotypes for SNP *j* + 1 [14]:

(5) $$e_{j+1}^{l+1}=e_{j}^{l+1}-x_{j}\left({\hat{a}}_{j}^{l+1}-{\hat{a}}_{j}^{l}\right).$$

Hereafter, the algorithm that uses equations (3), (4) and (5) will be referred to as “original residual updating”. In the original residual updating algorithm, updating of the residuals and obtaining the sum of cross-products of the residuals and genotypes of each individual are the most time-consuming steps [15]. As indicated by Legarra and Misztal [14], $x_{j}^{'}x_{j}$ can be calculated once and stored in memory. As a result, to compute ${\hat{a}}_{j}^{l+1}$ using equation (4), *m* + 1 multiplications are required. The number of multiplications can be reduced by first summing residuals ($e_{j}^{l+1}$) across animals with the same genotypes, and then multiplying each of those three sums by the appropriate genotype. Considering this, equation (4) can be rewritten as:

(6) $${\hat{a}}_{j}^{l+1}=\frac{\gamma_{j}^{'}f_{j}^{l+1}+n_{j}^{'}\gamma_{j}^{2}{\hat{a}}_{j}^{l}}{x_{j}^{'}x_{j}+\lambda_{j}},$$

where the **γ**_*j*_$\left(\gamma_{j}^{2}\right)\;$ is a vector that contains the (squared) centred and scaled values of the three genotypes that are present at locus *j*, $f_{j}^{l+1}$ is a 3 × 1 vector that contains the sum of the residuals for each genotype *i* at locus *j*, i.e. $f_{j}^{l+1}=\sum_{i}e_{i,j}^{l+1}$, and vector **n**_*j*_ contains the number of animals for each genotype at locus *j*. It should be noted here that $n_{j}^{'}\gamma_{j}^{2}=x_{j}^{'}x_{j}$, but the notation $n_{j}^{'}\gamma_{j}^{2}$ is introduced here to clarify implementation in the newly proposed algorithm, as will be shown later. Equation (6) involves only four multiplications and, thus, requires *m*-3 fewer multiplications than equation (4) (noting that all values for $n_{j}^{'}\gamma_{j}^{2}$ can be computed once and stored). Those *m*-3 multiplications are replaced by *m*-3 summations that are computationally less demanding than multiplications using standard Fortran functions. Hereafter, the algorithm that uses equations (5) and (6) will be referred to as “improved residual updating”.

Further reduction of the number of required computations is possible in the update for locus *j* + 1 by using the residual information that was already calculated in the update of locus *j*:

(7) $$f_{k,j+1}^{l+1}=h_{k,\;j+1}^{l+1}-N_{j+1,\;j}^{'}\gamma_{j}\left({\hat{a}}_{j}^{l+1}-{\hat{a}}_{j}^{l}\right),$$

where $f_{k,\;j+1}^{l+1}=\sum_{k}e_{k,\;j+1}^{l+1}$, i.e. a 3 × 1 vector that contains the sums of the residuals before updating locus *j* + 1 for each genotype *k* at locus *j* + 1 in iteration *l* + 1, and $N_{j+1,j}^{'}$ is a 3 × 3 matrix that contains the number of animals that have any of the nine combinations of genotypes at loci *j* and *j* + 1. Note that $N_{j+1,j}^{'}\gamma_{j}$ can be computed once and stored. The term $h_{k,j+1}^{l+1}$ is a vector that contains the sum of the residuals for each of the three genotypes *k* at locus *j* + 1. Each of these sums is computed from the sums of residuals (before updating) for each of the three genotypes *i* at locus *j* that were computed within groups of animals having genotype *k* at locus *j* + 1, i.e. $h_{k,\;j+1}^{l+1}=\sum_{k,\;j+1}\sum_{i}e_{i,\;j|k,\;j+1}^{l+1}$. Thus, for locus *j*, first 3 × 3 = 9 sums of residuals are calculated, one for each unique combination of genotypes at loci *j* and *j* + 1. These sums include the residuals before ${\hat{a}}_{j}^{l+1}$ is used to update them. The update at locus *j* is accounted for by the term $N_{j+1,\;j}^{'}\gamma_{j}\left({\hat{a}}_{j}^{l+1}-{\hat{a}}_{j}^{l}\right)$.

Implementing equation (7) in (6) yields:

(8) $${\hat{a}}_{j+1}^{l+1}=\frac{\gamma_{j+1}^{'}\left(h_{k,\;j+1}^{l+1}-\left(N_{j+1,\;j}^{'}\gamma_{j}\left({\hat{a}}_{j}^{l+1}-{\hat{a}}_{j}^{l}\right)\right)\right)+n_{j+1}^{'}\gamma_{j+1}^{2}{\hat{a}}_{j+1}^{l}}{x_{j+1}^{'}x_{j+1}+\lambda_{j+1}}.$$

The alternative proposed updating scheme to compute ${\hat{a}}_{j+1}^{l+1}$ involves applying equation (8) instead of (4) or (6) for locus *j* + 1. This updating scheme is hereafter referred to as “right-hand-side updating” (RHS-updating), since it essentially involves direct updating of the right-hand-sides of the model to estimate the SNP effects, without having to explicitly update the residuals every time a SNP effect is estimated.

Instead of explicitly updating residuals after computing the allele substitution effect, the change of the residuals is stored for each possible combination of genotypes at loci *j* and *j* + 1 as:

(9) $$\Delta e_{j+1,\;j}^{l+1}=\gamma_{j+1}1^{'}\left({\hat{a}}_{j+1}^{l+1}-{\hat{a}}_{j+1}^{l}\right)+1\gamma_{j}^{'}\left({\hat{a}}_{j}^{l+1}-{\hat{a}}_{j}^{l}\right),$$

where $\Delta e_{j+1,j}^{l+1}$ is a 3 × 3 matrix that contains updates to the residuals for each combination of genotypes at loci *j* and *j* + 1 after computing the allele substitution effects for those loci, and **1** is a vector of 1’s, such that both **γ**_*j* + 1_1′ and $1\gamma_{j}^{'}$ are 3 × 3 matrices. After computing ${\hat{a}}_{j}^{l+1}$ and ${\hat{a}}_{j+1}^{l+1}$, residuals for each combination of genotypes at loci *j* and *j* + 1 can be updated as:

(10) $$e_{k,\;j+1;\;i,\;j}^{l+1,\;j+2}=e_{k,\;j+1;\;i,\;j}^{l+1,j}-\Delta e_{k,\;j+1;\;i,\;j}^{l+1},$$

where $\Delta e_{k,\;j+1;\;i,\;j}^{l+1}$ is the element in $\Delta e_{j+1,\;j}^{l+1}$ that corresponds to genotype *k* at locus *j* + 1 and genotype *i* at locus *j*. Applying equations (9) and (10) for locus *j* and *j* + 1, finalizes the RHS-updating step, just like equation (5) finalizes the residual updating step.

A set of SNPs that is consecutively analysed using RHS-updating, is hereafter referred to as a “RHS-block”. It should be noted that for the first locus within an RHS-block, here referred to as locus *j*, there is no dependency on the previously evaluated locus and therefore equation (8) reduces to:

(11) $${\hat{a}}_{j}^{l+1}=\frac{\gamma_{j}f_{i,j}^{l+1}+n_{j}^{'}\gamma_{j}^{2}{\hat{a}}_{j}^{l}}{x_{j}^{'}x_{j}+\lambda_{j}}.$$

This can be interpreted as the initialization step where, first, the sums of residuals for all nine RHS-group are computed as $\sum_{i}e_{i,\;j|k,\;j+1}^{l+1}$ and, second, the sum of residuals for each genotype at locus *j* is computed as: $f_{i,\;j}^{l+1}=\sum_{i}\sum_{i}e_{i,\;j|k,\;j+1}^{l+1}$. Thus, the RHS-updating scheme for “RHS-blocks” of two loci involves the following steps:

1. Apply equation (11) for locus *j*,

2. Apply equation (8) for locus *j* + 1,

3. Apply equation (9) for locus *j* and *j* + 1, and

4. Apply equation (10) to update the residuals.

This implies that the number of operations for locus *j* is similar for the residual updating and RHS-updating algorithms. However, for locus *j* + 1, applying equation (8) requires only 20 summations and subtractions and 11 multiplications, compared to *m*-3 summations and 4 multiplications that are required when applying (6). This indicates that the total number of operations is drastically reduced by the RHS-updating algorithm.

**Figure 1:**
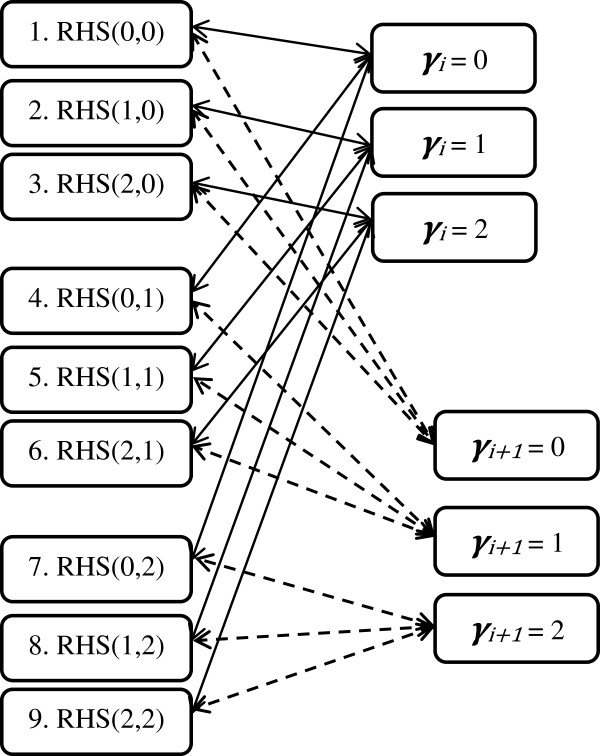
**Schematic overview of the groups defined within an RHS-block that includes two SNPs, in the RHS-updating scheme.** Groups are coded 1 to 9; RHS(*a*,*b*) represents the group within an RHS-block that combines individuals with genotype *a* at locus *j* and genotype *b* at locus *j* + 1.

Consider that for each pair of loci, groups of animals can be identified that have the same combination of genotypes at those two loci. With regard to the RHS-updating scheme, two important points should be noted. First, the groups within RHS-blocks can be coded such that each group code always contains the same genotypes on the first and second SNP. E.g., considering that there are 3^2^ groups. At locus *j* + 1, groups 1-3 contain genotype 0, groups 4-6 contain genotype 1, and groups 7-9 contain genotype 2. At locus *j*, groups 1, 4 and 7 contain genotype 0, groups 2, 5 and 8 contain genotype 1, and groups 3, 6 and 9 contain genotype 2. A schematic representation of this group coding within RHS-blocks is in Figure 1. Using such unique coding for the groups within RHS-blocks implies that genotypes do not need to be stored explicitly in memory, since they are stored implicitly through the group numbers. In the RHS-updating algorithm, the array that stores the group codes was stored as integer(2). In the residual updating algorithms, similarly, per locus and individual, the “codes” of the genotypes were stored in an integer(1) array while for each locus the actual values of the three genotypes were stored separately. In fact, the array storing the genotypes for the residual updating algorithm or the group codes for the RHS-updating algorithm are the largest arrays used in those algorithms, and therefore largely determine the total amount of RAM used. For residual updating, this array is of size *m* × *n* and was stored as integer(1) and the amount of RAM used is therefore expected to be proportional to *mn*. For RHS-updating, this array is of size *m* × *n*/*s*, where *s* is the number of SNPs per RHS-block and was stored as integer(2). The amount of RAM used with RHS-updating is therefore expected to be proportional to *2mn*/*s*. These simple formulas, adjusted to predict RAM use in Gb (Table 1), will be compared to empirically measured RAM use.

**Table 1:** Formulas to predict RAM requirement for original and improved residual updating, and for RHS-updating

**Algorithm**	**Predicted RAM requirements (Gb)**^**1**^
Original residual updating	*nm* × 10^−9^
Improved residual updating	*nm* × 10^−9^
RHS-updating	2 *nm* × 10^−9^/*s*

^1^*n* = number of loci; *m* = number of animals; *s* = number of loci included per RHS-block.

The second point that should be noted, is that within the RHS-updating scheme, the initialization step described in equation (10) is the most time-consuming and is recurrent every two loci. By applying the same principles, the RHS-updating scheme can be also applied to more than two loci consecutively. Increasing the number of loci per RHS-block may decrease the relative cost of the initialization step (10), but this will be eventually off-set by the exponential increase in the number of groups that is defined per RHS-block, which is equal to 3^s^, where *s* is the number of SNPs per RHS-block. When the number of loci per RHS-block increases, then at some stage the equivalent expression of equation (7) becomes computationally more demanding. The optimal value to be used for *s* likely depends on the number of individuals in the training data (*m*) and will be empirically derived in this study.

The three algorithms described above, are mathematically equivalent, in the sense that they estimate SNP effects using the same information. Thus, all three algorithms are expected to give the same results.

### Implementation of RHS-updating in Bayesian stochastic search variable selection

#### Model

The above updating schemes to estimate SNP effects were implemented for a model that is commonly referred to as Bayes SSVS (Stochastic Search Variable Selection) [16,17] that is solved using Gibbs sampling and implemented in a computer program written in Fortran 95. The genomic model is generally described as:

y=1μ+Xα+e,

where **y** contains phenotypic records, *μ* is the overall mean, **1** is a vector of 1’s, **X** is an *m* × *n* matrix that contains the scaled and centered genotypes of all individuals, **α** contains the (random) allele substitution effects for all loci, and **e** contains the random residuals. The specific parameterization of the genomic model that results in the Bayes SSVS model is described below.

#### Prior densities

The likelihood of the Bayes SSVS model conditional on all unknowns is assumed to be normal:

$$p\left(y_{i}|\mu ,\alpha ,\sigma_{e}^{2}\right)=N\left(y_{i}-\mu -x_{i}^{'}\alpha ,\sigma_{e}^{2}\right),$$

where **x**_*i*_ denotes the SNP genotypes of animal *i*. Definitions of the unknowns and their prior distributions are described hereafter.

The prior for *μ* was a constant. The residual variance $\sigma_{e}^{2}$ has a prior distribution of $p\left(\sigma_{e}^{2}\right)=\chi^{-2}\left(-2,0\right),$ which yields a flat prior.

The prior for *α*_*j*_ depends on the variance $\sigma_{\alpha }^{2}$ and the QTL indicator *I*_*j*_ = 1:

$$\alpha_{j}|\pi ,\sigma_{\alpha }^{2}=\left\{\begin{array}{c} \sim N\left(0,\frac{\sigma_{\alpha }^{2}}{100}\right)\;\mathrm{when}\;I_{j}=0\\ \sim N\left(0,\sigma_{\alpha }^{2}\right)\;\mathrm{when}\;I_{j}=1 \end{array}\right).$$

The prior distribution for the indicator variable *I*_*j*_  is:

$$p\left(I_{j}\right)=\mathrm{Bernoulli}\left(1-\pi \right),$$

where *π* was assigned a value of 0.999, and $\sigma_{\alpha }^{2}$ has the following prior distribution:

$$p\left(\sigma_{\alpha }^{2}\right)=\chi^{-2}\left(\nu_{\alpha },S_{\alpha }^{2}\right),$$

where *ν*_*α*_ is the degrees of freedom, set to 4.2 according to [12,15], and the scale parameter $S_{\alpha }^{2}$ is calculated as $S_{\alpha }^{2}=\frac{{\tilde{\sigma }}_{\alpha }^{2}\left(\nu_{\alpha }-2\right)}{\nu_{\alpha }}$ where ${\tilde{\sigma }}_{\alpha }^{2}$ is computed as [1]:

$${\tilde{\sigma }}_{\alpha }^{2}=\left(\frac{100}{100+\pi \left(1-100\right)}\right)\frac{\sigma_{a}^{2}}{n},$$

where *n* is the number of loci.

#### Conditional posterior densities

The conditional posterior density of α_*j*_ is:

$$N\left({\hat{\alpha }}_{j};\frac{\omega_{j}{\hat{\sigma }}_{e}^{2}}{x_{j}^{'}x_{j}+\lambda_{j}}\right),$$

where ${\hat{\alpha }}_{j}$ is the conditional mean of the allele substitution effect at locus *j*, whose computation was explained previously, $\lambda_{j}=\frac{\omega_{j}{\hat{\sigma }}_{e}^{2}}{{\hat{\sigma }}_{\alpha }^{2}}$, and

$$\begin{array}{ccc} \omega_{j}=1 & \mathrm{if} & I_{j}=1 \end{array}$$

$$\begin{array}{ccc} \omega_{j}=100 & \mathrm{if} & I_{j}=0. \end{array}$$

The conditional posterior density of $\sigma_{\alpha }^{2}$ is an inverse-χ^2^ distribution:

$$\sigma_{\alpha }^{2}|\alpha \sim \chi^{-2}\left(\nu_{\alpha }+n,S_{\alpha }^{2}+\omega^{'}{\hat{\alpha }}^{2}\right),$$

where ${\hat{\alpha }}^{2}$ is a vector with squares of the current estimates of the allele substitution effects of all loci, weighted by vector **ω**, which contains values of 1 or 100 for each locus.

Finally, the conditional posterior distribution of the QTL-indicator *I*_*j*_ was (following the notation in [19]):

$$\mathrm{Pr}\left(I_{j}=1\right)=\frac{f\left(r_{j}|I_{j}=1\right)\left(1-\pi \right)}{f\left(r_{j}|I_{j}=0\right)\pi +f\left(r_{j}|I_{j}=1\right)\left(1-\pi \right)},$$

where 1 − *π* (*π*) is the prior probability that *I*_*j*_ = 1 (*I*_*j*_ = 0), $r_{j}=x_{j}^{'}y^{*}+x_{j}^{'}x_{j}{\hat{\alpha }}_{j}$, where y* contains the conditional phenotypes as defined previously, and *f*(*r*_*j*_|*I*_*j*_ = δ), where δ is either 0 or 1, and is proportional to $\frac{1}{\sqrt{v}}e^{-\frac{r_{j}^{2}}{2v}}$, where $v={\left(x_{j}^{'}x_{j}\right)}^{2}\frac{\sigma_{\alpha_{j}}^{2}}{\omega_{j}}+x_{j}^{'}x_{j}\sigma_{e}^{2}$. It should be noted that *v* depends on *I*_*j*_ through its dependence on ω_*j*_, i.e. if *I*_*j*_ = 0 (*I*_*j*_ = 1) then ω_*j*_ = 100 (ω_*j*_ = 1).

Finally, the conditional posterior density of $\sigma_{e}^{2}$ is an inverse-χ^2^ distribution:

$$\sigma_{e}^{2}|e\sim \chi^{-2}\left(m-2,e^{'}e\right),$$

where *m* is the number of animals with records and **e** is a vector with the current residuals.

### Derivation of the optimal number of SNPs included per RHS-block

#### Simulated data - CPU time

To investigate to what extent the CPU time of the newly developed algorithms depended on the number of individuals included in the analysis, a dataset of 420 SNPs was simulated. The number of SNPs was limited to 420, to reduce total computation time, and this limited number of SNPs was sufficient to compare the relative computation time of the different algorithms because total CPU time scales linearly with the number of SNPs. Further details of the simulation are not included here because the aim of the subsequent analysis was only to compare CPU time. The size of the dataset was increased with steps of 500 individuals from 500 to 100 000 individuals and each dataset was analysed 11 times. The newly developed RHS-updating algorithm was used in nine of those analyses, using one to nine SNPs per RHS-block. The tenth analysis was an implementation using original residual updating. The eleventh analysis implemented improved residual updating. Each analysis was run for 900 iterations, and the CPU time for these 900 iterations was recorded.

#### Simulated data - RAM use

To investigate to what extent the required amount of RAM of the newly developed algorithm depended on the number of individuals in the analysis, a dataset of 50 000 SNPs was simulated. The number of individuals was increased in steps of 5000 individuals from 500 to 95 500 individuals. This number of SNPs and animals yielded a range of datasets with dimensions that corresponded to the size of currently used practical datasets and each dataset was analysed 11 times. These analyses involved the same models and settings as used to evaluate CPU time. Each analysis was run until the iterations started, i.e. when the maximum RAM requirement was reached, at which point the used RAM was recorded and the process was aborted. The maximum RAM requirement was measured by retrieving the process ID and then storing the RAM use for that process. Details of this procedure are provided in the Appendix 1.

All comparisons were run on a Windows XP-64 desktop pc with an Intel(R) Xeon(R) 64-bit CPU E5420 with a clock speed of 2.50 GHz. Comparisons on CPU time were also run on a Linux platform with an AMD Opteron 8431 64-bit CPU with a clock speed of 2.39 GHz running Ubuntu 12.04.3. The programs were compiled with the Intel® Fortran Compiler 11.0.075 for Windows and the Intel® Fortran Compiler 13.0.079 for Linux.

## Results

### CPU time

**Figure 2:**
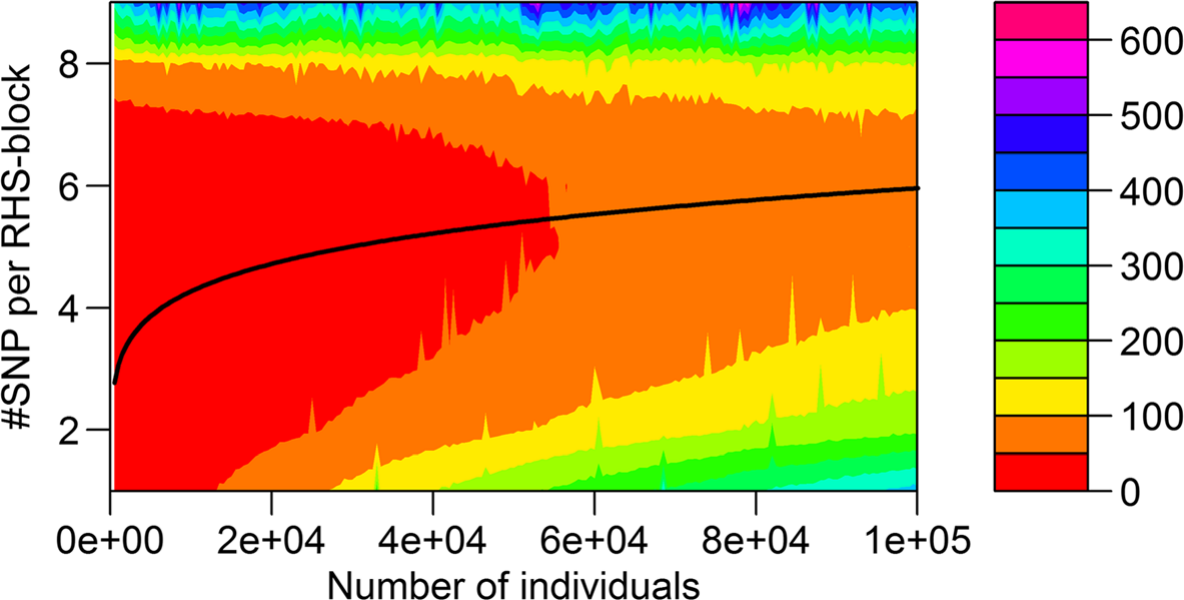
**CPU time on a Windows workstation for RHS-updating using different numbers of SNPs per RHS-block.** The reported time is for Gibbs chains of 900 iterations; the algorithm used one to nine SNPs per RHS-block, and the data contained 420 SNPs and an increasing number of individuals (500 to 100 000); the black line is the fitted curve through the number of SNPs that gave the minimum CPU time.

**Figure 3:**
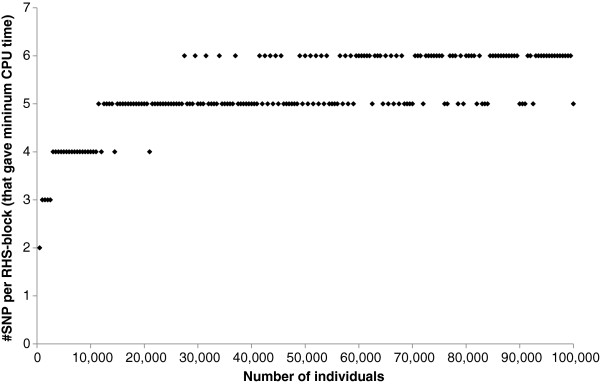
**The number of SNPs included per RHS-block in RHS-updating, that yielded the minimum computing time.** Minimum computing time was evaluated for an increasing number of individuals (500 to 100 000).

**Figure 4:**
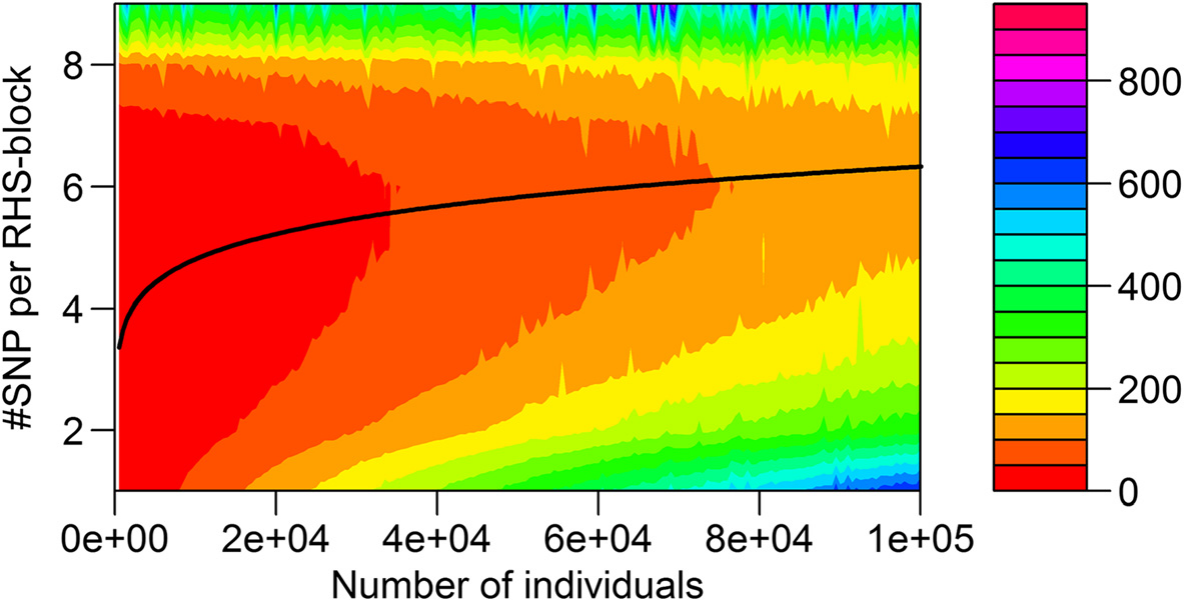
**CPU time on a Linux server for RHS-updating using different numbers of SNPs per RHS-block.** The reported time is for Gibbs chains of 900 iterations; the algorithm used one to nine SNPs per RHS-block, and the data contained 420 SNPs and an increasing number of individuals (500 to 100 000); the black line is the fitted curve through the number of SNP that gave the minimum CPU time.

The required CPU time on the Windows workstation for the RHS-updating algorithm depended strongly on the number of SNPs included per RHS-block (Figure 2). In all cases, including more than six SNPs per RHS-block resulted in a longer CPU time. Conversely, including less than four or five SNPs per RHS-block also resulted in a longer CPU time, especially when the number of individuals was large. The solid line in Figure 2 shows that the optimal number of SNPs included per RHS-block that results in the lowest CPU time, changed with the number of individuals in the analysis. This is further illustrated in Figure 3, where the actual number of SNPs that gave the minimum CPU time is plotted against the number of individuals in the analysis. Although there was a clear trend, across the number of individuals, especially with a larger number of individuals, there was no exact threshold when either five or six SNPs were included per RHS-block, which indicates that there was very little difference in CPU time when five or six SNPs were used. Similar trends in CPU time were observed when running the analysis on the Linux server, for which slightly longer CPU times were generally observed (Figure 4). Nevertheless, with our implementation of RHS-updating, it appears to be appropriate to include two SNPs when the number of individuals is less than 1000, three SNPs when it is between 1000 and 2500, four SNPs when it is between 2500 and 11 000, five SNPs when it is between 11 000 and 50 000, and six SNPs when it is between 50 000 and 100 000.

**Figure 5:**
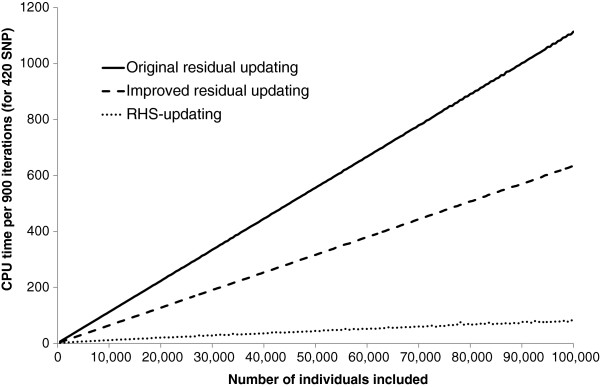
**CPU time on a Windows workstation using two residual updating schemes and RHS-updating.** CPU time for a Gibbs chain of 900 iterations was evaluated for 420 SNPs and an increasing number of individuals (500 to 100 000); the reported CPU time for the RHS-updating scheme is the minimum computing time of nine analyses that include one to nine SNPs per RHS-block, for each number of individuals.

The CPU time for the Gibbs chain using the optimal number of SNPs per RHS-block was compared to the CPU time of the original and improved residual updating schemes across the different numbers of animals included in the analysis (Figure 5). Compared to original residual updating, improved residual updating reduced CPU time on the Windows workstation by 35.3 to 43.3%, and RHS-updating reduced CPU time by 74.5 to 93.0%. Improvements in terms of CPU time were similar for the Linux server (results not shown). The required CPU time for pre-processing the data was slightly larger for the RHS-updating versus the residual updating algorithm. On the Windows workstation, for the dataset with 95 500 animals and 50 000 SNPs, the time to transform the SNP data into the (integer) coding, required 954 s and 1217 s, respectively, for the residual updating and the RHS-updating algorithm. These results show that, although most of the reduction in CPU time achieved by RHS-updating originated from evaluating SNPs within RHS-blocks rather than individually, at the same time, the first step to implement improved residual updating already makes an important contribution to the reduction in CPU time.

### RAM use

**Figure 6:**
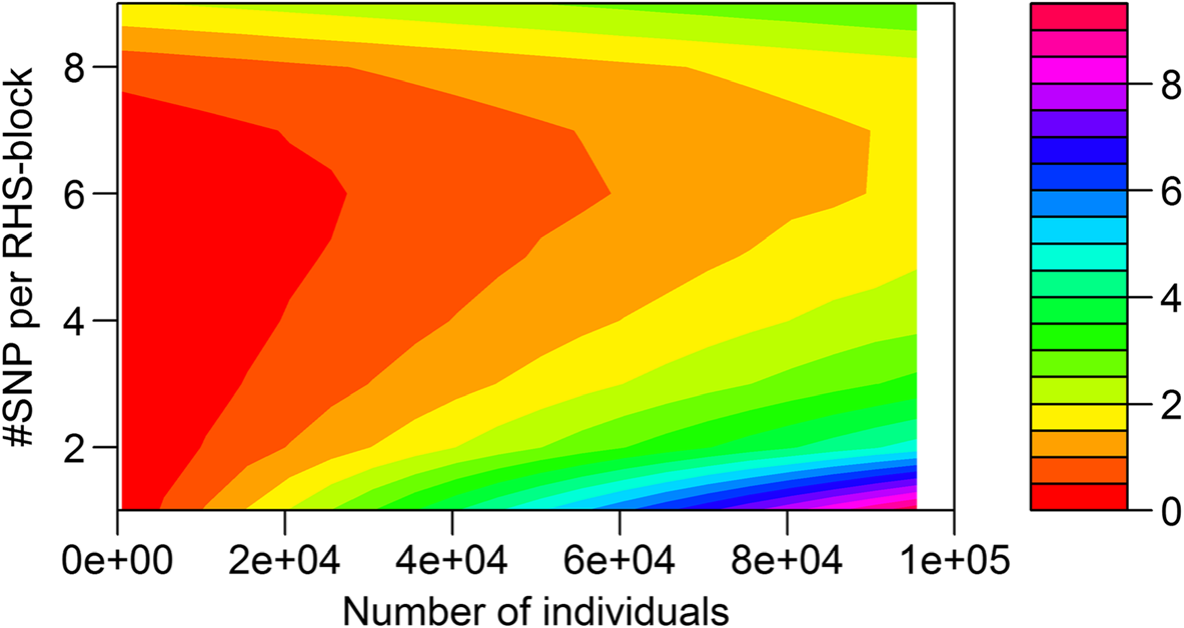
**RAM use (Gb) for RHS-updating with one to nine SNPs per RHS-block.** RAM use was evaluated for 50 000 SNPs and an increasing number of individuals (500 to 95 500).

The required amount of RAM for the RHS-updating algorithm also depended on the number of SNPs included per RHS-block (Figure 6). In nearly all cases, including more than six SNPs per RHS-block resulted in more RAM used. Conversely, including less than three to six SNPs per RHS-block also resulted in more RAM used, especially when the number of individuals was large. The pattern in RAM use (Figure 6), changed with the number of individuals in the dataset, in a pattern that was quite similar to that of the CPU time. This implies that choosing an optimal number of SNPs per RHS-block based on required CPU time, yields an algorithm that is also close to optimal in terms of RAM use.

**Figure 7:**
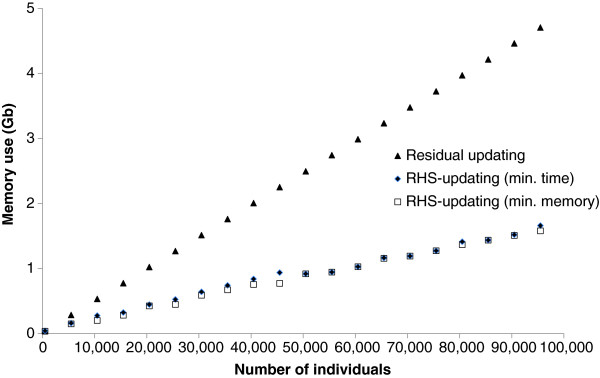
**RAM use (Gb) for residual updating versus RHS-updating.** For RHS-updating, the number of SNPs per RHS-block was set to the number that gave either the minimum computing time (min. time) or the minimum RAM requirement (min. RAM); RAM use was evaluated on a Windows workstation for a dataset with 50 000 SNPs and for an increasing number of individuals (500 to 95 500); note that only one curve is plotted for residual updating, because both residual updating algorithms require practically the same amount of RAM.

In Figure 7, the amount of RAM used is plotted for all three algorithms, for datasets with 50 000 SNPs and 500 to 95 500 individuals. Note that both residual updating algorithms required about the same amount of RAM, therefore only one curve was plotted for residual updating. In the case of RHS-updating, for each number of individuals, either the number of SNPs per RHS-block that gave the minimum CPU time or the number that gave the minimum amount of required RAM was used. Both sets of numbers of SNPs per RHS-block gave very similar answers. Compared to the residual updating schemes, the reduction in required RAM for RHS-updating ranged from 13.1 to 66.4%.

As shown in Figure 7, for a fixed number of SNPs, the RAM use of the residual updating and the RHS-updating algorithms were linearly related to the number of animals included. This agrees with the derived formulas for expected RAM use in Table 1. The ability of those equations to predict measured RAM use, was investigated by regressing measured RAM use on predicted RAM use for each method, based on datasets containing 50 000 SNPs and 500 to 95 500 animals. The results of those regressions are presented in Table 2 and show that the equations predicted RAM use with an R^2^ value of 1.0 in all cases. The intercepts of the regression generally had a positive value, indicating that the prediction equation missed only a small proportion of the used RAM. This value became substantial for the RHS-updating algorithm when the number of SNPs per RHS-block was equal to 7 or more because, e.g., the relative size of the array that stores the number of individuals for each RHS-group increases considerably when the number of RHS-blocks increases.

**Table 2:** Coefficients of the regression of measured on predicted RAM requirements for original and improved residual updating, and for RHS-updating

**Algorithm**	**Intercept**	**Slope**	**R**^**2**^
Original residual updating	0.014	0.983	1.000
Improved residual updating	0.014	0.983	1.000
RHS-updating (1)^1^	0.015	0.981	1.000
RHS-updating (2)	0.015	0.983	1.000
RHS-updating (3)	0.016	0.985	1.000
RHS-updating (4)	0.024	0.981	1.000
RHS-updating (5)	0.035	0.983	0.999
RHS-updating (6)	0.065	0.977	0.996
RHS-updating (7)	0.229	0.989	1.000
RHS-updating (8)	0.659	0.990	1.000
RHS-updating (9)	1.948	0.992	1.000

^1^The number between brackets indicates the number of SNPs per RHS-block.

## Discussion

Two alternative algorithms were presented that can be implemented in various genomic prediction models for fast computing of SNP effects. The algorithms replace the originally suggested residual updating algorithm [14], without affecting the results obtained. Differences in results between algorithms were similar to those within algorithms when using different random seeds and correlations between different sets of results were greater than 0.99 (results not shown). Both algorithms use the characteristic that only three different genotypes are observed for each SNP. Both algorithms can accommodate loci with more than three genotypes, but this may reduce or eliminate their benefit in terms of computing time over original residual updating schemes. The limitation on the number of genotypes per locus implies that imputed genotypes defined as gene contents cannot be used for individuals in the training data in the algorithm. Nevertheless, a simple transformation of gene contents to the most likely genotype overcomes this problem. This transformation could for instance be (on a 0-2 scale): genotypes ≤ 0.5 are set to 0, genotypes ≥ 1.5 are set to 2, and all other genotypes are set to 1. Such transformations for imputed genotypes in the training data are expected to have a minor impact on the estimated SNP effects, provided that the genotypes are imputed with reasonable accuracy. Using gene contents for selection candidates, i.e. individuals whose genetic merit is predicted using SNP effects estimated from the training data, is not inhibited by the proposed algorithms, because their predicted genetic merit can simply be obtained outside the algorithm by multiplying their gene contents with allele substitution effects that are estimated in the algorithm. The literature shows that for selection candidates, predictions differ when gene contents or the most likely genotypes are used [20].

The residual updating algorithms were implemented using standard (e.g. dot_product) Fortran functions. Computer-specific optimized libraries are available [21,22] that can considerably reduce the CPU time required for, e.g., vector and matrix multiplications [23]. Using such libraries may have a larger impact on CPU time for the residual updating algorithms than for the RHS-updating algorithm, since the former involves many more multiplications. However, even when using such optimized libraries, the RHS-updating algorithm is still expected to be considerably more efficient, because it drastically reduces the total number of required operations.

It should be noted that the RHS-updating scheme requires slightly more overhead in terms of computing time than the residual updating scheme, for instance to define the RHS-blocks and the group coding within those blocks. Once RHS-blocks and group coding are defined, they can be used in each iteration of the Gibbs chain. This may limit flexibility in the algorithm. For instance, one way to improve mixing of the Gibbs chains, may be to permute the order of evaluation of SNP effects between iterations. With the RHS-updating scheme, the order of evaluation of SNP effects within RHS-blocks must be the same throughout the Gibbs chain, such that group coding within RHS-blocks needs to be defined only once. Nevertheless, the order of evaluation of the RHS-blocks can still be permuted. Furthermore, to avoid that neighbouring SNPs are always evaluated in the same order, SNPs can be allocated to RHS-blocks at random.

The RHS-updating scheme not only considerably reduced computing time, by up to 93%, but also resulted in a reduction of the amount of RAM used of up to 66%. Due to the nature of the RHS-updating algorithm, computing time and RAM use are linearly related with the number of SNPs considered, similar to the residual updating algorithm. This implies that the relative benefit of using the RHS-updating algorithm compared to the residual updating algorithm is not affected by the number of SNPs included. In our implementation, which is written in Fortran 95, group codes within RHS-blocks were stored as an integer(2) variable, while the genotypes in the original implementation with residual updating were stored as an integer(1) variable. It should be noted that storing group codes as integer(1) would lead to a further reduction in RAM requirements of almost 50%, because the array that stores the group codes uses close to 100% of the RAM used by the algorithm. Storing group codes as integer(1), implies that the number of SNPs included per RHS-block should be equal to four or less, i.e. the maximum value an integer(1) variable can take is equal to 127, and including 4 (5) SNPs per RHS-block yields 3^4^ = 81 (3^5^ = 243) groups; the maximum value an integer(2) variable can take, is equal to 32 767. This means that a maximum of nine SNPs can be included per RHS-block, otherwise the group code must be stored as integer(4), i.e. including 9 (10) SNPs per RHS-block yields 3^9^ = 19 683 (3^10^ = 59 049) groups. Our results show that with the largest number of individuals considered (100 000), the optimal number of SNPs included per RHS-block was equal to six. This suggests that it is unlikely that a number of individuals in the data that justifies including more than nine SNPs per RHS-block, and therefore requires storing group codes as integer(4), is reached in the near future.

In our implementation of RHS-updating, each RHS-block containing *s* SNPs is assumed to contain all 3^*s*^ possible groups, which is most likely not always the case. Moreover, clever grouping of SNPs within RHS-block can reduce the observed number of groups within each RHS-block. Such redundancy could be used to further reduce computing time, but would also likely result in a more complicated algorithm.

## Conclusions

Two algorithms are presented to estimate SNP effects that can be implemented in a range of different genomic prediction models, as an alternative to the original residual updating scheme. The first alternative algorithm uses residual updating, here termed improved residual updating, and takes advantage of the characteristic that the predictor variables in the model (i.e. SNP genotypes) have only three possible values. The second alternative algorithm, here termed “RHS-updating”, extends the idea of improved residual updating across multiple SNPs. The improved residual updating algorithm achieved a reduction in computing time of 35.3 to 43.3%, but did not change the amount of RAM used, compared to the original residual updating scheme. The RHS-updating algorithm achieved a reduction in computing time of 74.5 to 93.0% and a reduction in RAM use of 13.1 to 66.4%, compared to the original residual updating scheme. Thus, the RHS-updating algorithm provides an interesting alternative to reduce both computing time and memory requirements.

## Appendix 1. Pseudo-code to measure maximum RAM requirement

The maximum RAM requirement was measured by retrieving the process ID and then storing the RAM use for this particular process. On the Windows OS, this was done using the following Fortran code:

PROGRAM GIBBS

USE DFPORT !Module that contains function “GETPID”

IMPLICIT NONE

INTEGER :: PROC_ID

CHARACTER(LEN = 47) :: SYS_CALL

…

PROC_ID = GET_PID() !Retrieve process ID of the current process

!Use DOS command “tasklist” to write RAM use to file “tasklist.txt”

SYS_CALL = "tasklist /fi ""PID eq ""  > tasklist.txt"

WRITE(SYS_CALL(23:29),'(i7)')PROC_ID

CALL SYSTEM(SYS_CALL)

…

END PROGRAM GIBBS

## Competing interests

The author declares that he has no competing interests.

## Authors’ contributions

MPLC has invented and developed the idea of RHS-block updating, implemented it in an algorithm, designed and performed the analyses and drafted the manuscript. The author read and approved the final manuscript.
